# Supplementary material for: Gerontology and Geriatrics in Undergraduate Nursing Education in Portugal and Spain: An Integrative and Comparative Curriculum Review
Source: Healthcare (Basel). 2024 Sep 6;12(17):1786. doi: 10.3390/healthcare12171786 (PMC11395543; doi:10.3390/healthcare12171786)
Supplement: Supplementary file 1 [file healthcare-12-01786-s001.zip › Supplementary_material_tableS3_qualityAppraisal.pdf]

Supplementary Materials Table S3 – Quality Appraisal

Citations

AlSenany, AlSaif (2014) 2
Bevil et al. (1988) 3
Brunner and Kada (2010) 4
Brunner and Kada (2010) 5
Chang and Do (2024) 6
Deschodt et al. (2009) 7
Ghaffari et al. (2016) 8
Hsieh and Chen (2018) 9
Koskinen et al., (2015) 10
Naidoo et al. (2020) 11
Nawagi et al. (2022) 12
Krichbaum et al. (2015) 13
Hsieh et al. (2018) 14
Tavares et al. (2021) 15
Wenxian Xu et al. (2024) 16
WHO Europe gerontological nursing curriculum (2003) 17
Wilson (2010) 18

AlSenany, AlSaif (2014)[1]

Category of study designs	Methodological quality criteria	Responses			
		Yes	No	Can't tell	Comments
Screening questions (for all types)	S1. Are there clear research questions?	X			
	S2. Do the collected data allow to address the research questions?	X			
	Further appraisal may not be feasible or appropriate when the answer is 'No' or 'Can't tell' to one or both screening questions.				
1. Qualitative	1.1. Is the qualitative approach appropriate to answer the research question?				
	1.2. Are the qualitative data collection methods adequate to address the research question?				
	1.3. Are the findings adequately derived from the data?				
	1.4. Is the interpretation of results sufficiently substantiated by data?				
	1.5. Is there coherence between qualitative data sources, collection, analysis and interpretation?				
2. Quantitative randomized controlled trials	2.1. Is randomization appropriately performed?				
	2.2. Are the groups comparable at baseline?				
	2.3. Are there complete outcome data?				
	2.4. Are outcome assessors blinded to the intervention provided?				
	2.5 Did the participants adhere to the assigned intervention?				
3. Quantitative non-randomized	3.1. Are the participants representative of the target population?				
	3.2. Are measurements appropriate regarding both the outcome and intervention (or exposure)?				
	3.3. Are there complete outcome data?				
	3.4. Are the confounders accounted for in the design and analysis?				
	3.5. During the study period, is the intervention administered (or exposure occurred) as intended?				
4. Quantitative descriptive	4.1. Is the sampling strategy relevant to address the research question?				
	4.2. Is the sample representative of the target population?				
	4.3. Are the measurements appropriate?				
	4.4. Is the risk of nonresponse bias low?				
	4.5. Is the statistical analysis appropriate to answer the research question?				
5. Mixed methods	5.1. Is there an adequate rationale for using a mixed methods design to address the research question?	X			
	5.2. Are the different components of the study effectively integrated to answer the research question?	X			
	5.3. Are the outputs of the integration of qualitative and quantitative components adequately interpreted?	X			
	5.4. Are divergences and inconsistencies between quantitative and qualitative results adequately addressed?	X			
	5.5. Do the different components of the study adhere to the quality criteria of each tradition of the methods involved?			X	

Bevil et al. (1988) [2]

JBI Critical Appraisal Checklist For Textual Evidence: Policy/Consensus Guidelines

Reviewer: Sara Brás Alves

Date: Junho/2024

Author: Bevil et al

Year:1988

Record Number: 2

	Yes	No	Unclear	Not applicable
1. Are the developers of the policy/ consensus guideline (and any allegiences/affiliations) clearly identified?	X	☐	☐	☐
2. Do the developers of the policy/ consensus guideline have standing in the field of expertise?	X	☐	☐	☐
3. Are appropriate stakeholders involved in developing the policy/guideline and do the conclusions drawn represent the views of their intended users?	X	☐	☐	☐
4. Are biases due to competing interests acknowledged and responded to?	☐	☐	X	☐
5. Are the processes of gathering and summarizing the evidence described?	X	☐	☐	☐
6. Is any incongruence with the extant literature/evidence logically defended?	☐	☐	☐	X
7. Are the methods used to develop recommendations described?	☐	☐	☐	X

Overall appraisal: Include X Exclude ☐

Brunner and Kada (2010) [3]

JBI Critical Appraisal Checklist For Textual Evidence: Expert Opinion

Reviewer: Sara Brás Alves____Date: Junho/2024

Author: Brunner and Kada______________________Year 2010 Record Number - 3

	Yes	No	Unclear	Not applicable
1. Is the source of the opinion clearly identified?	X	☐	☐	☐
2. Does the source of opinion have standing in the field of expertise?	☐	☐	X	☐
3. Are the interests of the relevant population the central focus of the opinion?	X	☐	☐	☐
4. Does the opinion demonstrate a logically defended argument to support the conclusions drawn?	X	☐	☐	☐
5. Is there reference to the extant literature?	X	☐	☐	☐
6. Is any incongruence with the literature/sources logically defended?	☐	☐	☐	X

Overall appraisal: Include X Exclude ☐ Seek further info ☐

Brunner and Kada (2010) [3]

JBI Critical Appraisal Checklist For Textual Evidence: Expert Opinion

Reviewer: Sara Brás Alves____Date: Junho/2024

Author: Brunner and Kada________________________Year 2010 Record Number - 3

	Yes	No	Unclear	Not applicable
1. Is the source of the opinion clearly identified?	X	☐	☐	☐
2. Does the source of opinion have standing in the field of expertise?	☐	☐	X	☐
3. Are the interests of the relevant population the central focus of the opinion?	X	☐	☐	☐
4. Does the opinion demonstrate a logically defended argument to support the conclusions drawn?	X	☐	☐	☐
5. Is there reference to the extant literature?	X	☐	☐	☐
6. Is any incongruence with the literature/sources logically defended?	☐	☐	☐	X

Overall appraisal: Include X Exclude ☐ Seek further info ☐

Chang and Do (2024)[4]

Category of study designs	Methodological quality criteria	Responses			
		Yes	No	Can't tell	Comments
Screening questions (for all types)	S1. Are there clear research questions?	X			
	S2. Do the collected data allow to address the research questions?	X			
	Further appraisal may not be feasible or appropriate when the answer is 'No' or 'Can't tell' to one or both screening questions.				
1. Qualitative	1.1. Is the qualitative approach appropriate to answer the research question?				
	1.2. Are the qualitative data collection methods adequate to address the research question?				
	1.3. Are the findings adequately derived from the data?				
	1.4. Is the interpretation of results sufficiently substantiated by data?				
	1.5. Is there coherence between qualitative data sources, collection, analysis and interpretation?				
2. Quantitative randomized controlled trials	2.1. Is randomization appropriately performed?				
	2.2. Are the groups comparable at baseline?				
	2.3. Are there complete outcome data?				
	2.4. Are outcome assessors blinded to the intervention provided?				
	2.5 Did the participants adhere to the assigned intervention?				
3. Quantitative non-randomized	3.1. Are the participants representative of the target population?				
	3.2. Are measurements appropriate regarding both the outcome and intervention (or exposure)?				
	3.3. Are there complete outcome data?				
	3.4. Are the confounders accounted for in the design and analysis?				
	3.5. During the study period, is the intervention administered (or exposure occurred) as intended?				
4. Quantitative descriptive	4.1. Is the sampling strategy relevant to address the research question?				
	4.2. Is the sample representative of the target population?				
	4.3. Are the measurements appropriate?				
	4.4. Is the risk of nonresponse bias low?				
	4.5. Is the statistical analysis appropriate to answer the research question?				
5. Mixed methods	5.1. Is there an adequate rationale for using a mixed methods design to address the research question?	X			
	5.2. Are the different components of the study effectively integrated to answer the research question?	X			
	5.3. Are the outputs of the integration of qualitative and quantitative components adequately interpreted?	X			
	5.4. Are divergences and inconsistencies between quantitative and qualitative results adequately addressed?	X			
	5.5. Do the different components of the study adhere to the quality criteria of each tradition of the methods involved?	X			

Deschodt et al. (2009) [5]

**Category of study designs**	**Methodological quality criteria**	**Responses**			
		Yes	No	Can't tell	Comments
Screening questions (for all types)	S1. Are there clear research questions?	X			
	S2. Do the collected data allow to address the research questions?	X			
	Further appraisal may not be feasible or appropriate when the answer is 'No' or 'Can't tell' to one or both screening questions.				
1. Qualitative	1.1. Is the qualitative approach appropriate to answer the research question?				
	1.2. Are the qualitative data collection methods adequate to address the research question?				
	1.3. Are the findings adequately derived from the data?				
	1.4. Is the interpretation of results sufficiently substantiated by data?				
	1.5. Is there coherence between qualitative data sources, collection, analysis and interpretation?				
2. Quantitative randomized controlled trials	2.1. Is randomization appropriately performed?				
	2.2. Are the groups comparable at baseline?				
	2.3. Are there complete outcome data?				
	2.4. Are outcome assessors blinded to the intervention provided?				
	2.5 Did the participants adhere to the assigned intervention?				
3. Quantitative non-randomized	3.1. Are the participants representative of the target population?				
	3.2. Are measurements appropriate regarding both the outcome and intervention (or exposure)?				
	3.3. Are there complete outcome data?				
	3.4. Are the confounders accounted for in the design and analysis?				
	3.5. During the study period, is the intervention administered (or exposure occurred) as intended?				
4. Quantitative descriptive	4.1. Is the sampling strategy relevant to address the research question?	X			
	4.2. Is the sample representative of the target population?	X			
	4.3. Are the measurements appropriate?	X			
	4.4. Is the risk of nonresponse bias low?			X	
	4.5. Is the statistical analysis appropriate to answer the research question?	X			
5. Mixed methods	5.1. Is there an adequate rationale for using a mixed methods design to address the research question?				
	5.2. Are the different components of the study effectively integrated to answer the research question?				
	5.3. Are the outputs of the integration of qualitative and quantitative components adequately interpreted?				
	5.4. Are divergences and inconsistencies between quantitative and qualitative results adequately addressed?				
	5.5. Do the different components of the study adhere to the quality criteria of each tradition of the methods involved?				

Ghaffari et al. (2016) [6]

Category of study designs	Methodological quality criteria	Responses			
		Yes	No	Can't tell	Comments
Screening questions (for all types)	S1. Are there clear research questions?	X			
	S2. Do the collected data allow to address the research questions?	X			
	Further appraisal may not be feasible or appropriate when the answer is 'No' or 'Can't tell' to one or both screening questions.				
1. Qualitative	1.1. Is the qualitative approach appropriate to answer the research question?	X			
	1.2. Are the qualitative data collection methods adequate to address the research question?	X			
	1.3. Are the findings adequately derived from the data?	X			
	1.4. Is the interpretation of results sufficiently substantiated by data?	X			
	1.5. Is there coherence between qualitative data sources, collection, analysis and interpretation?	X			
2. Quantitative randomized controlled trials	2.1. Is randomization appropriately performed?				
	2.2. Are the groups comparable at baseline?				
	2.3. Are there complete outcome data?				
	2.4. Are outcome assessors blinded to the intervention provided?				
	2.5 Did the participants adhere to the assigned intervention?				
3. Quantitative non-randomized	3.1. Are the participants representative of the target population?				
	3.2. Are measurements appropriate regarding both the outcome and intervention (or exposure)?				
	3.3. Are there complete outcome data?				
	3.4. Are the confounders accounted for in the design and analysis?				
	3.5. During the study period, is the intervention administered (or exposure occurred) as intended?				
4. Quantitative descriptive	4.1. Is the sampling strategy relevant to address the research question?				
	4.2. Is the sample representative of the target population?				
	4.3. Are the measurements appropriate?				
	4.4. Is the risk of nonresponse bias low?				
	4.5. Is the statistical analysis appropriate to answer the research question?				
5. Mixed methods	5.1. Is there an adequate rationale for using a mixed methods design to address the research question?				
	5.2. Are the different components of the study effectively integrated to answer the research question?				
	5.3. Are the outputs of the integration of qualitative and quantitative components adequately interpreted?				
	5.4. Are divergences and inconsistencies between quantitative and qualitative results adequately addressed?				
	5.5. Do the different components of the study adhere to the quality criteria of each tradition of the methods involved?				

Hsieh and Chen (2018) [7]

**Category of study designs**	**Methodological quality criteria**	**Responses**			
		Yes	No	Can't tell	Comments
Screening questions (for all types)	S1. Are there clear research questions?	X			
	S2. Do the collected data allow to address the research questions?	X			
	Further appraisal may not be feasible or appropriate when the answer is 'No' or 'Can't tell' to one or both screening questions.				
1. Qualitative	1.1. Is the qualitative approach appropriate to answer the research question?				
	1.2. Are the qualitative data collection methods adequate to address the research question?				
	1.3. Are the findings adequately derived from the data?				
	1.4. Is the interpretation of results sufficiently substantiated by data?				
	1.5. Is there coherence between qualitative data sources, collection, analysis and interpretation?				
2. Quantitative randomized controlled trials	2.1. Is randomization appropriately performed?				
	2.2. Are the groups comparable at baseline?				
	2.3. Are there complete outcome data?				
	2.4. Are outcome assessors blinded to the intervention provided?				
	2.5 Did the participants adhere to the assigned intervention?				
3. Quantitative non-randomized	3.1. Are the participants representative of the target population?				
	3.2. Are measurements appropriate regarding both the outcome and intervention (or exposure)?				
	3.3. Are there complete outcome data?				
	3.4. Are the confounders accounted for in the design and analysis?				
	3.5. During the study period, is the intervention administered (or exposure occurred) as intended?				
4. Quantitative descriptive	4.1. Is the sampling strategy relevant to address the research question?	X			
	4.2. Is the sample representative of the target population?			X	
	4.3. Are the measurements appropriate?	X			
	4.4. Is the risk of nonresponse bias low?			X	
	4.5. Is the statistical analysis appropriate to answer the research question?	X			
5. Mixed methods	5.1. Is there an adequate rationale for using a mixed methods design to address the research question?				
	5.2. Are the different components of the study effectively integrated to answer the research question?				
	5.3. Are the outputs of the integration of qualitative and quantitative components adequately interpreted?				
	5.4. Are divergences and inconsistencies between quantitative and qualitative results adequately addressed?				
	5.5. Do the different components of the study adhere to the quality criteria of each tradition of the methods involved?				

Koskinen et al., (2015) [8]

JBI Critical Appraisal Checklist For Systematic Reviews And Research Syntheses

Reviewer: Sara Brás Alves____Date: Junho/2024

Author: Koskinen et al__________________________Year 2015 Record Number - 8

	Question	Yes	No	Unclear	Not applicable
1.	Is the review question clearly and explicitly stated?	X	☐	☐	☐
2.	Were the inclusion criteria appropriate for the review question?	X	☐	☐	☐
3.	Was the search strategy appropriate?	X	☐	☐	☐
4.	Were the sources and resources used to search for studies adequate?	X	☐	☐	☐
5.	Were the criteria for appraising studies appropriate?	X	☐	☐	☐
6.	Was critical appraisal conducted by two or more reviewers independently?	X	☐	☐	☐
7.	Were there methods to minimize errors in data extraction?	X	☐	☐	☐
8.	Were the methods used to combine studies appropriate?	X	☐	☐	☐
9.	Was the likelihood of publication bias assessed?	X	☐	☐	☐
10.	Were recommendations for policy and/or practice supported by the reported data?	X	☐	☐	☐
11.	Were the specific directives for new research appropriate?	X	☐	☐	☐

Overall appraisal: Include X Exclude ☐ Seek further info ☐

Naidoo et al. (2020)[9]

**Category of study designs**	**Methodological quality criteria**	**Responses**			
		Yes	No	Can't tell	Comments
Screening questions (for all types)	S1. Are there clear research questions?	X			
	S2. Do the collected data allow to address the research questions?	X			
	Further appraisal may not be feasible or appropriate when the answer is 'No' or 'Can't tell' to one or both screening questions.				
1. Qualitative	1.1. Is the qualitative approach appropriate to answer the research question?				
	1.2. Are the qualitative data collection methods adequate to address the research question?				
	1.3. Are the findings adequately derived from the data?				
	1.4. Is the interpretation of results sufficiently substantiated by data?				
	1.5. Is there coherence between qualitative data sources, collection, analysis and interpretation?				
2. Quantitative randomized controlled trials	2.1. Is randomization appropriately performed?				
	2.2. Are the groups comparable at baseline?				
	2.3. Are there complete outcome data?				
	2.4. Are outcome assessors blinded to the intervention provided?				
	2.5 Did the participants adhere to the assigned intervention?				
3. Quantitative non-randomized	3.1. Are the participants representative of the target population?				
	3.2. Are measurements appropriate regarding both the outcome and intervention (or exposure)?				
	3.3. Are there complete outcome data?				
	3.4. Are the confounders accounted for in the design and analysis?				
	3.5. During the study period, is the intervention administered (or exposure occurred) as intended?				
4. Quantitative descriptive	4.1. Is the sampling strategy relevant to address the research question?				
	4.2. Is the sample representative of the target population?				
	4.3. Are the measurements appropriate?				
	4.4. Is the risk of nonresponse bias low?				
	4.5. Is the statistical analysis appropriate to answer the research question?				
5. Mixed methods	5.1. Is there an adequate rationale for using a mixed methods design to address the research question?	X			
	5.2. Are the different components of the study effectively integrated to answer the research question?	X			
	5.3. Are the outputs of the integration of qualitative and quantitative components adequately interpreted?			X	
	5.4. Are divergences and inconsistencies between quantitative and qualitative results adequately addressed?			X	
	5.5. Do the different components of the study adhere to the quality criteria of each tradition of the methods involved?			X	

Nawagi et al. (2022) [10]

**Category of study designs**	**Methodological quality criteria**	**Responses**			
		Yes	No	Can't tell	Comments
Screening questions (for all types)	S1. Are there clear research questions?	X			
	S2. Do the collected data allow to address the research questions?	X			
	Further appraisal may not be feasible or appropriate when the answer is 'No' or 'Can't tell' to one or both screening questions.				
1. Qualitative	1.1. Is the qualitative approach appropriate to answer the research question?				
	1.2. Are the qualitative data collection methods adequate to address the research question?				
	1.3. Are the findings adequately derived from the data?				
	1.4. Is the interpretation of results sufficiently substantiated by data?				
	1.5. Is there coherence between qualitative data sources, collection, analysis and interpretation?				
2. Quantitative randomized controlled trials	2.1. Is randomization appropriately performed?				
	2.2. Are the groups comparable at baseline?				
	2.3. Are there complete outcome data?				
	2.4. Are outcome assessors blinded to the intervention provided?				
	2.5 Did the participants adhere to the assigned intervention?				
3. Quantitative non-randomized	3.1. Are the participants representative of the target population?				
	3.2. Are measurements appropriate regarding both the outcome and intervention (or exposure)?				
	3.3. Are there complete outcome data?				
	3.4. Are the confounders accounted for in the design and analysis?				
	3.5. During the study period, is the intervention administered (or exposure occurred) as intended?				
4. Quantitative descriptive	4.1. Is the sampling strategy relevant to address the research question?	X			
	4.2. Is the sample representative of the target population?			X	
	4.3. Are the measurements appropriate?	X			
	4.4. Is the risk of nonresponse bias low?	X			
	4.5. Is the statistical analysis appropriate to answer the research question?	X			
5. Mixed methods	5.1. Is there an adequate rationale for using a mixed methods design to address the research question?				
	5.2. Are the different components of the study effectively integrated to answer the research question?				
	5.3. Are the outputs of the integration of qualitative and quantitative components adequately interpreted?				
	5.4. Are divergences and inconsistencies between quantitative and qualitative results adequately addressed?				
	5.5. Do the different components of the study adhere to the quality criteria of each tradition of the methods involved?				

Krichbaum et al. (2015) [11]

**Category of study designs**	**Methodological quality criteria**	**Responses**			
		Yes	No	Can't tell	Comments
Screening questions (for all types)	S1. Are there clear research questions?	X			
	S2. Do the collected data allow to address the research questions?	X			
	Further appraisal may not be feasible or appropriate when the answer is 'No' or 'Can't tell' to one or both screening questions.				
1. Qualitative	1.1. Is the qualitative approach appropriate to answer the research question?				
	1.2. Are the qualitative data collection methods adequate to address the research question?				
	1.3. Are the findings adequately derived from the data?				
	1.4. Is the interpretation of results sufficiently substantiated by data?				
	1.5. Is there coherence between qualitative data sources, collection, analysis and interpretation?				
2. Quantitative randomized controlled trials	2.1. Is randomization appropriately performed?				
	2.2. Are the groups comparable at baseline?				
	2.3. Are there complete outcome data?				
	2.4. Are outcome assessors blinded to the intervention provided?				
	2.5 Did the participants adhere to the assigned intervention?				
3. Quantitative non-randomized	3.1. Are the participants representative of the target population?				
	3.2. Are measurements appropriate regarding both the outcome and intervention (or exposure)?				
	3.3. Are there complete outcome data?				
	3.4. Are the confounders accounted for in the design and analysis?				
	3.5. During the study period, is the intervention administered (or exposure occurred) as intended?				
4. Quantitative descriptive	4.1. Is the sampling strategy relevant to address the research question?				
	4.2. Is the sample representative of the target population?				
	4.3. Are the measurements appropriate?				
	4.4. Is the risk of nonresponse bias low?				
	4.5. Is the statistical analysis appropriate to answer the research question?				
5. Mixed methods	5.1. Is there an adequate rationale for using a mixed methods design to address the research question?	X			
	5.2. Are the different components of the study effectively integrated to answer the research question?	X			
	5.3. Are the outputs of the integration of qualitative and quantitative components adequately interpreted?	X			
	5.4. Are divergences and inconsistencies between quantitative and qualitative results adequately addressed?	X			
	5.5. Do the different components of the study adhere to the quality criteria of each tradition of the methods involved?	X			

Hsieh et al. (2018) [12]

JBI Critical Appraisal Checklist For Systematic Reviews And Research Syntheses

Reviewer: Sara Brás Alves____Date: Junho/2024

Author: Hsieh et al_____________________________Year 2018 Record Number - 12

	Yes	No	Unclear	Not applicable
12. Is the review question clearly and explicitly stated?	X	☐	☐	☐
13. Were the inclusion criteria appropriate for the review question?	X	☐	☐	☐
14. Was the search strategy appropriate?	X	☐	☐	☐
15. Were the sources and resources used to search for studies adequate?	X	☐	☐	☐
16. Were the criteria for appraising studies appropriate?	X	☐	☐	☐
17. Was critical appraisal conducted by two or more reviewers independently?	X	☐	☐	☐
18. Were there methods to minimize errors in data extraction?	X	☐	☐	☐
19. Were the methods used to combine studies appropriate?	X	☐	☐	☐
20. Was the likelihood of publication bias assessed?	X	☐	☐	☐
21. Were recommendations for policy and/or practice supported by the reported data?	X	☐	☐	☐
22. Were the specific directives for new research appropriate?	X	☐	☐	☐

Overall appraisal: Include X Exclude ☐ Seek further info ☐

Tavares et al. (2021) [13]

**Category of study designs**	**Methodological quality criteria**	**Responses**			
		Yes	No	Can't tell	Comments
Screening questions (for all types)	S1. Are there clear research questions?	X			
	S2. Do the collected data allow to address the research questions?	X			
	Further appraisal may not be feasible or appropriate when the answer is 'No' or 'Can't tell' to one or both screening questions.				
1. Qualitative	1.1. Is the qualitative approach appropriate to answer the research question?				
	1.2. Are the qualitative data collection methods adequate to address the research question?				
	1.3. Are the findings adequately derived from the data?				
	1.4. Is the interpretation of results sufficiently substantiated by data?				
	1.5. Is there coherence between qualitative data sources, collection, analysis and interpretation?				
2. Quantitative randomized controlled trials	2.1. Is randomization appropriately performed?				
	2.2. Are the groups comparable at baseline?				
	2.3. Are there complete outcome data?				
	2.4. Are outcome assessors blinded to the intervention provided?				
	2.5 Did the participants adhere to the assigned intervention?				
3. Quantitative non-randomized	3.1. Are the participants representative of the target population?				
	3.2. Are measurements appropriate regarding both the outcome and intervention (or exposure)?				
	3.3. Are there complete outcome data?				
	3.4. Are the confounders accounted for in the design and analysis?				
	3.5. During the study period, is the intervention administered (or exposure occurred) as intended?				
4. Quantitative descriptive	4.1. Is the sampling strategy relevant to address the research question?	X			
	4.2. Is the sample representative of the target population?	X			
	4.3. Are the measurements appropriate?	X			
	4.4. Is the risk of nonresponse bias low?	X			
	4.5. Is the statistical analysis appropriate to answer the research question?	X			
5. Mixed methods	5.1. Is there an adequate rationale for using a mixed methods design to address the research question?				
	5.2. Are the different components of the study effectively integrated to answer the research question?				
	5.3. Are the outputs of the integration of qualitative and quantitative components adequately interpreted?				
	5.4. Are divergences and inconsistencies between quantitative and qualitative results adequately addressed?				
	5.5. Do the different components of the study adhere to the quality criteria of each tradition of the methods involved?				

Wenxian Xu et al. (2024) [14]

Category of study designs	Methodological quality criteria	Responses			
		Yes	No	Can't tell	Comments
Screening questions (for all types)	S1. Are there clear research questions?	X			
	S2. Do the collected data allow to address the research questions?	X			
	Further appraisal may not be feasible or appropriate when the answer is 'No' or 'Can't tell' to one or both screening questions.				
1. Qualitative	1.1. Is the qualitative approach appropriate to answer the research question?				
	1.2. Are the qualitative data collection methods adequate to address the research question?				
	1.3. Are the findings adequately derived from the data?				
	1.4. Is the interpretation of results sufficiently substantiated by data?				
	1.5. Is there coherence between qualitative data sources, collection, analysis and interpretation?				
2. Quantitative randomized controlled trials	2.1. Is randomization appropriately performed?				
	2.2. Are the groups comparable at baseline?				
	2.3. Are there complete outcome data?				
	2.4. Are outcome assessors blinded to the intervention provided?				
	2.5 Did the participants adhere to the assigned intervention?				
3. Quantitative non-randomized	3.1. Are the participants representative of the target population?				
	3.2. Are measurements appropriate regarding both the outcome and intervention (or exposure)?				
	3.3. Are there complete outcome data?				
	3.4. Are the confounders accounted for in the design and analysis?				
	3.5. During the study period, is the intervention administered (or exposure occurred) as intended?				
4. Quantitative descriptive	4.1. Is the sampling strategy relevant to address the research question?	X			
	4.2. Is the sample representative of the target population?	X			
	4.3. Are the measurements appropriate?	X			
	4.4. Is the risk of nonresponse bias low?	X			
	4.5. Is the statistical analysis appropriate to answer the research question?	X			
5. Mixed methods	5.1. Is there an adequate rationale for using a mixed methods design to address the research question?				
	5.2. Are the different components of the study effectively integrated to answer the research question?				
	5.3. Are the outputs of the integration of qualitative and quantitative components adequately interpreted?				
	5.4. Are divergences and inconsistencies between quantitative and qualitative results adequately addressed?				
	5.5. Do the different components of the study adhere to the quality criteria of each tradition of the methods involved?				

WHO Europe gerontological nursing curriculum (2003) [15]

JBI Critical Appraisal Checklist For Textual Evidence: Policy/Consensus Guidelines

Reviewer: Sara Brás Alves Date: Junho/2024

Author: World Health Organization. Regional Office for Europe Year:2003 Record Number: 15

	Yes	No	Unclear	Not applicable
7. Are the developers of the policy/ consensus guideline (and any allegiences/affiliations) clearly identified?	X	☐	☐	☐
8. Do the developers of the policy/ consensus guideline have standing in the field of expertise?	X	☐	☐	☐
9. Are appropriate stakeholders involved in developing the policy/guideline and do the conclusions drawn represent the views of their intended users?	X	☐	☐	☐
10. Are biases due to competing interests acknowledged and responded to?	☐	☐	X	☐
11. Are the processes of gathering and summarizing the evidence described?	☐	☐	☐	X
12. Is any incongruence with the extant literature/evidence logically defended?	☐	☐	☐	X
13. Are the methods used to develop recommendations described?	☐	☐	☐	X

Overall appraisal: Include X Exclude ☐ Seek further info

Wilson (2010) [16]

JBI Critical Appraisal Checklist For Textual Evidence: Policy/Consensus Guidelines

Reviewer: Sara Brás Alves Date: Junho/2024

Author: Laurie Wilson Year:2010 Record Number: 15

	Yes	No	Unclear	Not applicable
1. Is the source of the opinion clearly identified?	X	☐	☐	☐
2. Does the source of opinion have standing in the field of expertise?	X	☐	☐	☐
3. Are the interests of the relevant population the central focus of the opinion?	X	☐	☐	☐
4. Does the opinion demonstrate a logically defended argument to support the conclusions drawn?	X	☐	☐	☐
5. Is there reference to the extant literature?	X	☐	☐	☐
6. Is any incongruence with the literature/sources logically defended?	☐	☐	☐	X

Overall appraisal: Include X Exclude ☐ Seek further info

1. AlSenany, S.; AlSaif, A.A. Gerontology Course in the Nursing Undergraduate Curricula. *Rev Esc Enferm USP* **2014**, *48*, 1074–1081, doi:10.1590/S0080-623420140000700016.

2. Bevil, C.A.; Fields, S.D.; Davis, D.K. Toward a Core Curriculum for Interdisciplinary Geriatric Care. *Gerontol Geriatr Educ* **1988**, *8*, 201–215, doi:10.1300/J021V08N03_17.

3. Brunner, E.; Kada, O. Professionalisation of Gerontological Nursing — The Development of an International Online Gerontological Master Degree Programme. *Nurse Educ Today* **2010**, *30*, 779–783, doi:10.1016/J.NEDT.2010.03.001.

4. Chang, H.K.; Do, Y.J. A Spark of Change: Developing an Innovative Gerontological Nursing Intervention Mapping Initiative for Training and Education (IGNITE). *BMC Med Educ* **2024**, *24*, 1–9, doi:10.1186/S12909-024-05240-5/TABLES/5.

5. Deschodt, M.; De Casterlé, B.D.; Milisen, K. Gerontological Care in Nursing Education Programmes. *J Adv Nurs* **2010**, *66*, 139–148, doi:10.1111/J.1365-2648.2009.05160.X.

6. Ghaffari, F.; Dehghan-Nayeri, N.; Navabi, N.; Seylani, K. Evaluation of the Master's Curriculum for Elderly Nursing: A Qualitative Study. *Clin Interv Aging* **2016**, *11*, 1333, doi:10.2147/CIA.S109004.

7. Hsieh, P.L.; Chen, C.M. Nursing Competence in Geriatric/Long Term Care Curriculum Development for Baccalaureate Nursing Programs: A Systematic Review. *Journal of Professional Nursing* **2018**, *34*, 400–411, doi:10.1016/J.PROFNURS.2018.05.006.

8. Koskinen, S.; Salminen, L.; Stolt, M.; Leino-Kilpi, H. The Education Received by Nursing Students Regarding Nursing Older People: A Scoping Literature Review. *Scand J Caring Sci* **2015**, *29*, 15–29, doi:10.1111/SCS.12135.

9. Naidoo, K.; Waggie, F.; Van Wyk, J.M. A Review of Geriatric Care Training in the Undergraduate Nursing and Medical Curricula at the University of KwaZulu-Natal, South Africa. *Afr J Health Prof Educ* **2020**, *12*, 130, doi:10.7196/AJHPE.2020.V12I3.1349.

10. Nawagi, F.; Mukisa, J.; Najjuma, J.N.; Nabirye, R.C. "We Are Never Taught Anything about the Elderly." Establishing the Gap in Elderly Health Care Competencies in Nursing Education in Uganda. *BMC Nurs* **2022**, *21*, 1–11, doi:10.1186/S12912-022-00936-9/FIGURES/1.

11. Krichbaum, K.; Kaas, M.J.; Wyman, J.F.; Van Son, C.R. Facilitated Learning to Advance Geriatrics: Increasing the Capacity of Nurse Faculty to Teach Students About Caring for Older Adults. *Gerontologist* **2015**, *55*, S154–S164, doi:10.1093/GERONT/GNU165.

12. Hsieh, P.L.; Chen, C.M. Geriatric Nursing and Long Term Care Content in Baccalaureate Nursing Programs in Taiwan. *Int J Gerontol* **2018**, *12*, 52–56, doi:10.1016/J.IJGE.2017.04.001.

13. Tavares, J.; de Lurdes Almeida, M.; Duarte, S.F.C.; Apóstolo, J. Older Adult Care in Nursing Education: How Have Curricula Been Developed? *Nurse Educ Pract* **2021**, *50*, 102947, doi:10.1016/J.NEPR.2020.102947.

14. Xu, W.; Xie, X.; Fan, B.; Huang, Y.; Zhu, X.; Yang, Y. Developing Geriatric Nursing Micro-Credentials for Undergraduate Nursing Students Based on Training Objectives: A

Modified Delphi Study. *Nurse Educ Pract* **2024**, *76*, 103910, doi:10.1016/J.NEPR.2024.103910.

15. WHO Europe Gerontological Nursing Curriculum WHO European Strategy for Continuing Education for Nurses and Midwives 2003. **2003**.

16. Wilson, L.D. The American Association of Colleges of Nursing's Geriatric Nursing Education Consortium. *J Gerontol Nurs* **2010**, *36*, 14–17, doi:10.3928/00989134-20100528-01.
