# Supplementary material for: Gerontology and Geriatrics in Undergraduate Nursing Education in Portugal and Spain: An Integrative and Comparative Curriculum Review
Source: Healthcare (Basel). 2024 Sep 6;12(17):1786. doi: 10.3390/healthcare12171786 (PMC11395543; doi:10.3390/healthcare12171786)
Supplement: Supplementary file 1 [file healthcare-12-01786-s001.zip › Supplementary_material_tableS4_StudiesExtraction.pdf]

Supplementary Materials Table S5 – Studies data extraction

Author(s)/ Year	Local	Title	Sample sie and characteristics	Study design	Study objetive	Key findings	Conclusions
**K Naidoo, F Waggie, J M van Wyk (2020)[1]**	South Africa	A review of geriatric care training in the undergraduate nursing and medical curricula at the University of KwaZulu-Natal, South Africa	The study involved a descriptive exploratory approach through document review and semi-structured interviews with health professions educators. Key informants (n=5) involved in teaching and curriculum development were interviewed.	Descriptive exploratory study	To investigate the undergraduate medical and nursing curricula at a South African university regarding geriatric care training and explore possible learning opportunities to enhance health professions education in geriatric care.	Challenges found: Lack of independent assessment of geriatric topics, time constraints, lack of confidence of educators to implement interprofessional education (IPE), limited coverage and lack of discrete assessment of geriatric care competencies in both nursing and medical curricula. Implications: Inadequate training of health professionals in geriatric care may lead to suboptimal care for older adults, potentially affecting their quality of life and health outcomes.	The study highlighted the need to expand teaching and assessment relevant to the care of older adults in nursing and medical training programs, ensure greater concordance between the two disciplines, and enhance interprofessional education to prepare graduates for providing coordinated and quality care to older adults. Faculty development and additional resources are required to improve geriatric care training in both programs.

Author(s)/ Year	Local	Title	Sample sie and characteristics	Study design	Study objetive	Key findings	Conclusions
**K Naidoo, F Waggie, J M van Wyk (2020)**	South Africa	A review of geriatric care training in the undergraduate nursing and medical curricula at the University of KwaZulu-Natal, South Africa	The study involved a descriptive exploratory approach through document review and semi-structured interviews with health professions educators. Key informants (n=5) involved in teaching and curriculum development were interviewed.	Descriptive exploratory study	To investigate the undergraduate medical and nursing curricula at a South African university regarding geriatric care training and explore possible learning opportunities to enhance health professions education in geriatric care.	Challenges found: Lack of independent assessment of geriatric topics, time constraints, lack of confidence of educators to implement interprofessional education (IPE), limited coverage and lack of discrete assessment of geriatric care competencies in both nursing and medical curricula. Implications: Inadequate training of health professionals in geriatric care may lead to suboptimal care for older adults, potentially affecting their quality of life and health outcomes.	The study highlighted the need to expand teaching and assessment relevant to the care of older adults in nursing and medical training programs, ensure greater concordance between the two disciplines, and enhance interprofessional education to prepare graduates for providing coordinated and quality care to older adults. Faculty development and additional resources are required to improve geriatric care training in both programs.
**Kathleen Krichbaum, Merrie J. Kaas,**	USA	Facilitated Learning to Advance Geriatrics:	The article focuses on nurse faculty participating in the program, but	The article describes the development and	The main objective is to increase the expertise of nurse faculty in geriatrics to improve the	The main challenges identified include the lack of focus on care of older adults in nursing	The article emphasizes the importance of enhancing gerontological/geriatric nursing education to

**Jean F. Wyman, and Catherine R. Van Son (2015)**		Increasing the Capacity of Nurse Faculty to Teach Students About Caring for Older Adults	specific sample size and characteristics are not provided.	implementation of the Facilitated Learning to Advance Geriatrics program, which is designed to enhance the capacity of nurse faculty to teach students about caring for older adults.	education of nursing students in caring for older adults.	programs, the traditional undervaluing of geriatrics content, and the shortage of faculty with expertise in geriatrics. These challenges have implications for the quality of care provided to older adults by the future healthcare workforce.	address the growing needs of the aging population. It highlights the necessity of increasing faculty expertise in geriatrics to effectively prepare nursing students to care for older adults.
**Chang and Do (2024)**	South Korea	Developing an Innovative Educational Program in Gerontological Nursing	30 individuals from Gyeongsangnam-do, Korea, including registered hospital nurses and clinical nursing graduate students with at least one year of clinical experience	Single-group pretest-posttest design for the pilot program intervention.	To assess the effectiveness of an educational program in gerontological nursing for practicing nurses and graduate students.	The study identified main challenges in providing geriatric nursing education, including ageism, attitudes towards older adults, and knowledge gaps. Implications include the need for transformative behavioral changes, enhanced empathy, and the application of	The research highlights the importance of adaptive and empathetic educational models in preparing nursing professionals for the future of healthcare, emphasizing the significance of storytelling, person-centered care, and technological advancements in

						innovative educational strategies to improve care for older adults	gerontological nursing education.
**Fatemeh Ghaffari et al. (2016)**	Iran	Evaluation of the Master's curriculum for elderly nursing: a qualitative study	The study population included students, graduates, and professors of geriatric nursing at the Master of Science level. Sixteen interviews were conducted until data saturation was reached.	Qualitative study	To identify the advantages and weaknesses of a geriatric nursing program at Tehran University of Medical Sciences.	The main challenges identified were "motivation to enter geriatric nursing", "lack of employment groundwork", and "lack of practical implementation of the curriculum." The lack of suitable working fields and practical training opportunities were highlighted as major obstacles for students and graduates in the field of geriatric nursing. Implications include the need for restructuring administrative systems, providing job opportunities, and improving education facilities to enhance practical implementation of the curriculum.	Efforts to restructure administrative and employment systems can shift the focus from degree orientation to learning and increased expertise in geriatric nursing. Providing job opportunities and specialization by wards for elderly care can ensure the future of geriatric work. Regular evaluation of education centers and curriculum outcomes is recommended to enhance the quality of training provided to geriatric nurses.

**Pei-Lun Hsieh, Ching-Min Chen (2018)**	Taiwan	Geriatric Nursing and Long Term Care Content in Baccalaureate Nursing Programs in Taiwan	The study included 14 general universities and 11 universities of science and technology offering bachelor nursing education programs in Taiwan.	Descriptive research design	To survey the current GN or LTC curriculum in baccalaureate nursing programs in Taiwan.	The GN course content included topics such as the aging process, communication, ethical issues, health promotion, nutrition, and dementia care. LTC courses focused on the LTC system, policy, management of LTC institutions, ethical issues, and various LTC services like day care, home care, and LTC facilities. The study identified a need for more comprehensive education in geriatric nursing and LTC to meet the growing demands of the aging population in Taiwan.<br><br>**Main challenges found + implications:** The study highlighted the lack of clear articulation and standards for gerontology education in	The survey findings emphasized the importance of recognizing GN and LTC education by incorporating stand-alone courses and interdisciplinary education in nursing curricula. It suggested that these courses should be integrated into curricular design and innovations to ensure nursing students acquire the necessary competence for future healthcare challenges in geriatric and long-term care settings.

						Taiwan, indicating a need for improved training and education for healthcare professionals to provide high-quality care to the elderly and disabled population.	
**M. Deschodt et al. (2009)**	Belgium	Gerontology in nursing programmes	A descriptive cross-sectional mail survey of 17 baccalaureate nursing education programmes in Flanders, Belgium, conducted in 2007.	Descriptive cross-sectional mail survey	To identify the coverage of gerontological care in baccalaureate nursing education programmes and assess the barriers to incorporating gerontological care aspects into curricula.	Main challenges found: Variability in gerontology courses, clinical placements, and expertise among programmes. Barriers included lack of interest in care for older people, lack of gerontology-	The study emphasizes the importance of formulating gerontology-related competencies, establishing a standard curriculum for gerontology, and investing in role models to strengthen gerontology content in nursing

						related competencies in curricula, and negative perceptions of gerontological care.<br><br>Implications:<br>Highlighted the need for increased gerontology content in nursing education to better prepare nurses for caring for older patients.	education programmes. It underscores the necessity of enhancing educators' expertise and addressing barriers to improve geriatric healthcare and ensure high-quality care for older patients.
**AlSenany S, AlSaif AA (2014)**	Saudi Arabia	Gerontology course in the nursing undergraduate curricula	Convenience sample of nursing faculty members from three universities in Saudi Arabia. Five focus groups were conducted, each consisting of 6-8 members, including two teachers from each specialty: medical surgical nursing, fundamental	Qualitative study using focus groups to explore the perceived status of gerontological nursing education in the undergraduate nursing curricula.	To discuss the status of gerontological nursing education in the undergraduate nursing curricula in Saudi Arabia and identify challenges and implications for improving gerontology teaching.	Challenges found: Disparity between educational needs of gerontological nursing and current curricula, lack of appropriate clinical experiences, and insufficient gerontological nurse educators.<br><br>Implications: Need for suitable clinical practice settings, specialist educators with positive	The study emphasizes the importance of integrating gerontology nursing courses into the nursing curriculum to bridge the gap between theory and practice, improve students' knowledge and attitudes towards older people, and prepare graduates to provide quality care for the aging population. Nursing educators are encouraged to evaluate and adjust their curricula regularly to meet

			nursing, and community health nursing.			attitudes towards older people, and innovative approaches to educate nurses on older adults' specific needs	the educational needs of gerontological nursing and enhance healthcare delivery for older adults.
**Wenxian Xu et al. (2024)**	China	Developing geriatric nursing micro-credentials for undergraduate nursing students based on training objectives: A modified Delphi study	Expert group discussion involved 13 participants, and a three-round Delphi survey included 15 nursing specialists in China.	Delphi study	To develop geriatric nursing micro-credentials for undergraduate nursing students based on standardized training objectives and specify the learning goals and course modules for each specific micro-credential.	**Main challenges found:** Negative attitudes towards older adults, poor perceptions of geriatric care among nursing students, and the gap between the demand for care of older people and the lack of professional geriatric nurses.<br><br>**Implications:** Reforming geriatric nursing education can improve students' knowledge of aging, promote confidence and willingness to engage in geriatric nursing, and	The development of geriatric nursing micro-credentials can provide a pathway for training geriatric nursing personnel, meet the market demand for geriatric nursing talent, and improve the quality of nursing education to prepare students as backups for fully credentialed geriatric caregivers.

						enhance the quality of care for older adults.	
**P.-L. Hsieh, C.-M. Chen (2018)**	Taiwan	Nursing Competence in Geriatric/Long Term Care Curriculum Development	The review included 18 articles, comprising 16 quantitative and 2 qualitative studies, focusing on nursing students in BSN programs.	The systematic review analyzed the impact of Geriatric Nursing and Long Term Care courses on nursing students' competence through a synthesis of quantitative and qualitative studies.	The objective was to assess the effectiveness of including Geriatric and Long Term Care content in BSN programs in enhancing nursing students' competence in providing care for the elderly population.	Main challenges found: The review identified challenges related to faculty preparation, course offerings, and the integration of clinical placements into the curriculum to adequately prepare nursing students for caring for older and disabled individuals.<br><br>Implications: By enhancing nursing students' competence in Geriatric/Long Term Care, the education system can better prepare future nurses to meet the healthcare needs of the aging population, including older workers requiring specialized care.	The systematic review highlighted the importance of developing Geriatric/Long Term Care curriculum in BSN programs to provide a strong foundation for nursing students in caring for the elderly and disabled. It emphasized the need for faculty preparation, practical clinical experience, and a comprehensive curriculum design to foster competent nursing students capable of meeting the challenges of an aging population

**João Tavares et al. (2021)**	Portugal	Older adult care in nursing education: How have curricula been developed?	The study involved 18 nursing schools in Portugal, with data collected through an online survey between May and October 2017.	Cross-sectional, descriptive study	The study aimed to analyze gerontology education in Portuguese Bachelor of Science in Nursing (BSN) programs, focusing on identifying gerontology-related contents, analyzing barriers to incorporating aged care content, and assessing nursing faculty preparedness in teaching gerontology.	Main challenges found: Barriers to incorporating gerontology content included the negative image of gerontological nursing, overloaded curriculum, lack of role models/preceptors, and lack of standards for gerontology practice competencies.<br><br>Implications: The study emphasized the importance of addressing these challenges to enhance gerontological nursing education and improve care for older adults.	The study highlighted the heterogeneous nature of gerontology education in nursing curricula across Portugal, with a need for standardization and enhancement of gerontology content. Developing a standard gerontology curriculum and competencies can positively impact older adult care and influence the next generation of nurses.
**Eva Brunner and Olivia Kada (2010)**	Austria	Professionalisation of gerontological nursing — The development of an international	Not applicable (descriptive article)	Descriptive article outlining the development of the GEROM programme	To address the need for adequate nursing curricula in the gerontological field through an international	The main challenges identified include the varying accreditation procedures and cultural differences inhibiting the implementation of a	The Bologna process offers an opportunity to improve gerontological nursing education by developing comprehensive programmes that address

		online gerontological master degree programme			online master degree programme	joint degree. Implications for older workers include the importance of institutional commitment and communication between partners for a successful process.	the challenges faced by older workers in the healthcare system 1.
**Sanna Koskinen et al., (2015)**	Finland	The education received by nursing students regarding nursing older people: a scoping literature review	66 research articles from 17 different countries	Scoping literature review	To describe and analyze empirical studies evaluating nurses' education regarding nursing older people	The main challenges identified included varying levels of validity and trustworthiness in the studies, with a need for more well-designed studies focusing on specific learning outcomes and factors influencing the implementation of nursing education for older people. Implications include the need for improved education strategies to enhance students' competence in caring for the elderly and to promote gerontological	The review emphasized the importance of addressing the educational needs of nursing students in caring for older people to ensure quality care for the elderly. It highlighted the necessity for more comprehensive and well-designed studies to guide educational strategies and improve the competence of nursing students in this specialized area

						nursing as a career choice.	
**Laurie Dodge Wilson / 2010**	USA	The American Association of Colleges of Nursing's Geriatric Nursing Education Consortium	Not specified	Descriptive overview of the GNEC project and its impact on nursing education.	To improve the quality of nursing care for older adults by enhancing geriatric content in nursing curricula.	The GNEC project has successfully supported faculty in integrating geriatric content into nursing courses, with many schools revising senior-level courses and some creating standalone geriatrics courses. Challenges included the need for faculty to have access to evidence-based resources and support from colleagues. The implications of these findings are improved education for nursing students in geriatric care.	The GNEC project has been effective in creating a cadre of nursing faculty capable of enhancing geriatric nursing education. Ongoing evaluation will continue to assess the impact of the project on nursing curricula.
**Catherine A. Bevil; Suzanne D. Fields; D. Karl Davis (1988)**	USA	Toward a Core Curriculum for Interdisciplinary Geriatric Care	Not specified	The article presents a conceptual framework for developing a core curriculum for interdisciplinar y geriatric care	The main objective is to propose a comprehensive core curriculum that addresses the interdisciplinary nature of geriatric care and enhances the knowledge and skills of healthcare	The main challenges identified in geriatric care include the need for interdisciplinary collaboration, specialized training in gerontology, and the integration of geriatric principles into healthcare	The authors conclude that implementing a core curriculum for interdisciplinary geriatric care is essential for meeting the complex needs of older adults and improving the overall quality of geriatric healthcare services. They

				based on the authors' expertise and experience in the field.	professionals working with older adults.	education. The implications for older workers include improved quality of care, better outcomes, and enhanced interdisciplinary communication and teamwork.	emphasize the importance of ongoing education and training in gerontology for healthcare professionals to provide optimal care for older adult patients.
**Nawagi et al. (2022)**	Uganda	We are never taught anything: a qualitative study on the gaps in geriatric nursing competencies in Uganda	The study included faculty members training nurses at certificate, diploma, and degree levels, in-service nurses from various regions in Uganda, and key informants from nursing leadership positions in Uganda.	93 nurses	Qualitative study using document review, focus group discussions (FGDs), and key informant interviews (KIIs). Study objective: To identify gaps in geriatric nursing competencies in nursing education in Uganda and explore potential solutions to address these gaps.	Main challenges found: Lack of geriatric nursing modules and competencies in most nursing training curricula in Uganda, leading to a deficiency in knowledge and skills among nurses in caring for the elderly.<br><br>Implications for nursing care: The absence of geriatric nursing education hinders the ability of nurses to provide quality care to the growing elderly population in Uganda, potentially	The study highlights the urgent need to integrate geriatric nursing competencies into nursing education curricula in Uganda to better equip nurses in providing comprehensive care to the elderly. Recommendations include the development of specific modules, training sessions, and continuous education programs focused on geriatric nursing to address the identified gaps and improve the quality of care for older adults in Uganda.

						compromising the health outcomes of older adults.	
**WHO Regional Office for Europe (2003)**		WHO Europe Gerontological Nursing Curriculum	Not applicable	Curriculum development	To provide a structured curriculum for the education of nurses in gerontological nursing. Results and key findings: The curriculum outlines competencies, teaching strategies, assessment methods, and the importance of lifelong learning for gerontological nurses.	Challenges identified may include the need for specialized knowledge and skills in caring for older adults, as well as the importance of promoting evidence-based practice. Implications for nursing care include the need for nurses to empower older people and their carers, as well as the practice of effective interpersonal and communication skills in caring for older adults.	The WHO Europe Gerontological Nursing Curriculum serves as a guide for educators in developing programs to train nurses in gerontological nursing. It emphasizes the importance of specialized knowledge, communication skills, and evidence-based practice in providing quality care for older adults. By addressing these key areas, nurses can better meet the unique needs of the aging population and improve the quality of care provided to older adults.
