# Supplementary material for: Gerontology and Geriatrics in Undergraduate Nursing Education in Portugal and Spain: An Integrative and Comparative Curriculum Review
Source: Healthcare (Basel). 2024 Sep 6;12(17):1786. doi: 10.3390/healthcare12171786 (PMC11395543; doi:10.3390/healthcare12171786)
Supplement: Supplementary file 1 [file healthcare-12-01786-s001.zip › Supplementary_material_tableS5_NursingSchools_dataExtraction.pdf]

Supplementary Materials Table S6 – Nursing Schools Data Extraction

Institution (Country)	Curriculum document (year course)	Depth of geriatric care content (brief summary)	Key findings/notes
**Escola Superior De Saúde De Bragança (Portugal)**<br><br>**Https://Portal3.Ipb.Pt/Index.Php/Pt/Guiaects/Cursos/Licenciaturas/Curso?Cod_Escola=7015&Cod_Curso=9500**	Course unit:<br>Gerontological and geriatric nursing<br>(1st year/2nd semester – 3 ECTS)	1 introduction: definition of basic terms and characterisation of the ageing process. The phenomenon of population ageing.<br>2. Biological and psychosocial<br>3. Bio-psycho-social changes associated with the ageing process.<br>4. Stereotypes and prejudices in Gerontology.<br>5. Specificities inherent to good practice in gerontological/geriatric care.<br>6. Policies/support networks (health and/or social) for the elderly	At the end of the course unit, students should be able to: 1. Identify the phenomenon of population ageing from a historical, cultural and social perspective; 2. Describe the different biological and psychosocial theories attempting to explain the natural ageing process; 3. Describe the structural and functional biological changes, as well as the psychosocial changes, that characterise the process of natural ageing; 4. Differentiate normal ageing processes from pathological deviations; 5. Understand the specific characteristics of good gerontological/geriatric care. Differentiate the normal processes of natural ageing from pathological deviations; 5. Understand the specificities inherent in good gerontological/geriatric care practice; 6. Identify the policies/networks supporting the elderly population
**E. U. De Enfermería De**	Course unit:<br>Geriatric and gerontological nursing	Introduction: demographic aspects.<br>Sociological profile of the over 65s in Spain.	

**Zamora/Salamanca**<br><br>**(Spain)**<br><br>**Https://Guias.Usal.Es/Node/201846**	(3rd year/1st semester – 6 ECTS)	Block i: general aspects of geriatric nursing and gerontology.<br>-concepts: gerontology/ geriatrics.<br>-different situations of old age: frailty.<br>-ageing: structural and physiological changes.<br>-theories of ageing.<br>Block ii: comprehensive approach to the geriatric patient. Global geriatric assessment.<br>-physical or clinical assessment.<br>-functional assessment.<br>-cognitive or mental assessment.<br>-social assessment.<br>Block iii: preventive and health promotion activities.<br>-health education: vaccinations, exercise...<br>-general daily life care<br>Block iv: most frequent pathologies in the elderly: nursing action.<br>-general aspects of illnesses in the elderly:<br>-geriatric syndromes: immobility, pressure ulcers, falls, dementia, delirium, constipation, incontinence, polypharmacy<br>-most important diseases in the elderly<br>Block v: resources<br>-levels of care<br>-social resources<br>-supportive products (technical aids)	

**Escola Superior de Enfermagem do Porto**<br><br>**https://estudar.esenf.pt/elementor-4688/**	Course unit:<br>Adult and elderly health<br>(1st year/1st semester – 6 ECTS)	Historical perspective of adult health;<br>Theories and concepts; Biological, psychological and social characteristics of the adult;<br>Epidemiological transition;<br>Health risk management;<br>Diseases preventable by vaccination in adults;<br>Adult vaccination schedule;<br>Healthcare-associated infections;<br>Occupational risks;<br>Promotion and prevention of accidents and co-morbidities;<br>Assessment of worker health;<br>Aging: History, concepts and theories;<br>Demography and epidemiology of aging in Portugal;<br>Aging as a developmental transition;<br>Factors that influence the health status of the elderly;<br>Geriatric syndromes;<br>Integrated Continuing Care;<br>The impact of terminal illness and death on the individual and family;<br>Euthanasia, dysthanasia and orthothanasia;<br>Family care;<br>Adult and Geriatric/Gerontological assessment.<br>Nursing interventions to promote adult health and active aging.	Recognize the characteristics of the development of adults and elderly people;<br>Identify the possible influence of professional factors on workers' health;<br>Know labor legislation, particularly that relating to Safety, Hygiene and Health of workers;<br>Identify and list the physiological changes related to the aging process;<br>Describe the vaccination schedule for adults and elderly people;<br>Identify changes related to the sexuality of adults and elderly people;<br>Construct and identify the cultural and social meaning of the dying process;<br>Recognize and cite the Ministry of Health programs aimed at the adult and elderly population;<br>Plan health promotion and disease prevention actions for the adult and elderly population;<br>Develop skills in the areas of autonomy and decision-making to solve adult and elderly health problems.
**Universidade de Santiago de Compostela**	Course unit:<br>Life Cycle Nursing. Geriatrics<br>(3rd year/2nd semester – 4.5 ECTS)	I.-Basics Module Of Geriatrics And Gerontology:<br>Topic 1. Conceptual bases<br>Topic 2. Ageing of populations<br>Topic 3. Demography of old age<br>Topic 4. Gerontological evaluation<br>Topic 5. Cognitive assessment<br>Topic 6. psychoaffective assessment<br>Topic 7: Social valuation<br><br>II.-Module Ii. Geriatrics	Know the demographic, social and health impact of population ageing.<br>Know the levels of assistance for the elderly.<br>Know the ageing process to establish the limits between ageing characteristics and disease in the elderly.<br>Identify the factors that contribute to or alter health in the elderly.<br>Know the geriatric patient widely and deeply and assess the real needs of the sick, elderly person.

		Topic 1.-Anatomical-functional changes in aging. Impacts on health<br>Topic 2. Pharmacology in the elderly<br><br>Geriatric syndromes:<br>Topic 1.-Cognitive impairment/Dementia/Acute Confusional Syndrome<br>Topic 2.-Infections<br>Topic 3. Pressure ulcers<br>Topic 4.-Falls, Post-fall syndrome, Immobilization syndrome<br>Topic 5. -Urinary incontinence	Caring for the elderly and their family, teaching them behavioural guidelines to prevent illness and recover health.<br>Plan nursing care, considering the mechanisms that cause pathologies in old age, their manifestations and evolution.<br>Integration into the health team for the planning, executing and evaluating the care plan for the elderly.
**Escola Superior De Enfermagem De Coimbra**<br><br>**Https://Www.Esenfc.Pt/Pt/Courses/100001/Discipline/1000113020**	Course unit:<br>Elderly nursing and geriatrics<br>(2nd year/1st semester – 3 ECTS)	1 ageing and society<br>- current social configurations and demographic trends<br>- support networks and social support for older people and families<br>2. Ageing: concepts and theories<br>- the ageing process<br>- changes in primary ageing<br>3. Geriatric syndromes: implications for practice<br>- frailty<br>- postural instability / falls<br>- malnutrition<br>- sleep disorders<br>- infections<br>- sphincter incontinence<br>- cognitive and communication impairment<br>3.1 available technologies and services that favour the safety and protection of older people/carers<br>4. Principles of care for older people with advanced illness and at the end of life<br>5. Comprehensive geriatric assessment: nursing intervention<br>6. Violence/maltreatment of older people<br>7. Active ageing and nursing intervention strategies:	Recognise the challenges and opportunities associated with the demographic, epidemiological and social transition at international and national levels.<br>2. Identify and list the physiological, psychological, cultural and environmental changes related to the ageing process.<br>3. Describe the problems of geriatric syndromes and their impact on the individual, family/informal carer and community.<br>4. Develop competences in the areas of autonomy and decision-making to solve health problems for older people.<br>5. Plan health promotion and disease prevention actions for the older population.<br>6. Understand the barriers that older people and their families may experience when accessing healthcare and/or managing their treatment regime.

		- promotion of healthy lifestyles and health surveillance<br>- therapeutic regime management	
**Universidad Complutense De Madrid**	Course unit:<br>Community nursing II:<br>Topic 20: care for the elderly: + healthy aging + care for patients in fragile/dependent situations<br>(3rd year/1st semester – 6 ECTS)<br><br>Https://enfermeria.ucm.es/file/enfermeria-comunitaria-2-22-23?ver	Acquire the skills for developing the functions and activities of the nursing professional in primary health care, applying the principles of health promotion, prevention and care of prevalent health problems in the community, both at an individual and to population groups.	Acquire the skills for developing the functions and activities of the nursing professional in primary health care, applying the principles of health promotion, prevention and care of prevalent health problems in the community, both at an individual and to population groups.
	Course unit:<br>Nursing of old age, palliative care and pain management<br><br>(4rd year/1st semester – 6 ECTS)<br>https://enfermeria.ucm.es/file/enfermeria-vejez-22-23?ver	- demographics of ageing and its socio-health repercussions. The biological process of ageing. Theories of ageing. Manifestations of ageing in the organism of the elderly. 4.- comprehensive geriatric assessment. 5.- atypical presentation of diseases in the elderly. 6.- concept and description of the major geriatric syndromes. 7. Study of the major geriatric syndromes (dementia, frailty, gait disorders, falls and fractures, dysphagia, incontinence and other alterations in excretion, depression and isolation). 8.- most prevalent health problems in the elderly: stroke and neurodegenerative diseases, cardiovascular problems (ischaemic heart disease and heart failure), respiratory problems. 9.- ethical problems in the care of the elderly. 10.- physiopathology of pain. 11.- types of pain. 12.- pain treatment and care of the person with pain. 13. 13.- concept of palliative care. 14.- the patient in a palliative situation. Causes. 15.- palliative care in the various symptoms of the terminally ill patient. 16. Duelling	Understand the characteristics of the ageing process and associated health problems in order to be able to establish specific care plans, highlighting pain and end-of-life care.<br>Understand the characteristics of the ageing process and associated health problems in order to be able to establish specific care plans, highlighting pain and end-of-life care.<br>Respond to the needs of the elderly, after specific assessment, and in situations of illness, disability, and dependence, establish communication strategies with the elderly patient and his/her family to facilitate the expression of their concerns and interests. Develop strategies for accompanying bereavement. Understand the biological changes associated with the ageing process and their repercussions on health. Identify the most frequent health problems in the elderly and the establishment of specific care plans. Develop competencies in the care of people in the terminal phase and emotional support for the patient and the family.
**Escola Superior De Saúde –**	Course unit:<br>adult and elderly nursing<br>(2nd year/1st semester – 6 ECTS)	1. The phenomenon of ageing and longevity in adults and the elderly: concepts, theories,	1. Describe the phenomenon of ageing and longevity in adults and the elderly;

**Universidade Dos Açores**<br><br>**Https://Ess.Uac.Pt/Disciplinas/78299-Enfermagem-Do-Adulto-E-Idoso/**		biological, psychosocial and cultural factors/implications<br>2. Strategies to promote healthy ageing and longevity with quality - from data collection to diagnostic decision-making, prescribing nursing interventions and evaluating results (basic nursing tools)<br>2.1 rights of adults and the elderly - preventing stereotypes attributed to the ageing process and the elderly<br>2.2 work, unemployment, retirement and leisure<br>2.3 accident prevention<br>2.4 prevention of ill-treatment<br>2.5 urinary and intestinal elimination (bowel problems - constipation)<br>2.6 physical exercise<br>2.7 prevention of complications from immobility, dampness and pressure ulcers<br>2.8 sexuality<br>2.9 sleep and rest<br>2.10 relationship/communication with people with visual and hearing impairments and dementia<br>2.11. Spirituality and religiosity<br>3. Community resources and social policies - their implications for nursing practice.	2. Describe strategies to promote healthy ageing in adults and the elderly, and family/carers to promote longevity with quality;<br><br>3. Identify the implications of existing policies and resources in the community
**Universidad De La Laguna**<br><br>**Universidad De La Laguna - Título Aplicación O Servicio (Ull.Es)**	Course unit:<br>clinical nursing i - gerontogeriatric nursing<br>(2nd year/1st semester – 6 ECTS)	Didactic unit 1. Nursing assistance to adults with health problems: clinical nursing<br>Topic 1. Chronic diseases. The hospital environment. Hospitalized patient care<br>Topic 2. Nursing care for the surgical patient: preoperative, intraoperative, postoperative<br>Topic 3. Nursing care for patients with acute pain<br>Didactic unit 2. Nursing of the elderly<br>Topic 4. Old age and aging processes. Health problems in the elderly<br>Topic 5. Nursing care for the elderly. Comprehensive geriatric assessment of the elderly	The student will know how to delimit the object of study of clinical and gerontogeriatric nursing, focusing its foundations on the development of holistic nursing.<br>- the student will be able to plan nursing care for adults and the elderly by applying the work method and the universal language or taxonomies of nursing.<br>- the student will be able to perform basic nursing techniques and procedures safely, effectively and making rational use of resources.<br>- the student will show a reflective and critical attitude towards the various phenomena that occur

		Topic 6. Aging and dependency. Nursing care for patients experiencing loss, grief, and death<br>Didactic unit 3. Workshops and seminars<br>Workshop 1. Basic clinical techniques in nursing<br>- workshop 2. Approach to adults with acute pain<br>- workshop 3. Nursing assessment of the elderly<br>- workshop 4. Strategies to promote healthy aging<br>- seminar 1. Clinical nursing and the patient in the hospital environment<br>- seminar 2. The approach of the dependent person and the nurse case manager<br>Didactic unit 4. Simulation practices<br>Simulated practice 1. Parenteral administration of medication (intramuscular/intradermal/subcutaneous/intravenous)<br>- simulated practice 2. Bladder catheterization<br>- simulated practice 3. Nasogastric catheterization<br>- simulated practice 4. Basic techniques for wound healing<br>- simulated practice 5. Oxygen administration and secretion aspiration technique<br>- simulated practice 6. Placement of peripheral venous catheter and serum therapy	in today's society and their influence on clinical and gerontogeriatric nursing.<br>- the student will be able to show independence in delving into new content in the field of clinical and gerontogeriatric nursing.
**Universidade Da Madeira – Escola De Enfermagem**<br><br>**(Portugal)**<br><br>**Https://Www.Uma.Pt/Ensino/1o-Ciclo/Licenciatura-Em-Enfermagem/10464/?Contentid=10464**	Course unit:<br>Nursing ii - nursing for the elderly<br>(1st year/2nd semester – 3 ECTS)	1 - the aging process and theoretical perspectives.<br>2 - bio-psycho-social-cultural and spiritual implications.<br>2.1. - stereotypes and prejudices.<br>3 - social, health policies and community resources.<br>4 - promotion of healthy aging and prevention of changes.<br>5 - autonomy and empowerment.<br>6 - nurse intervention in the independence-dependence continuum aiming at a better quality of life.<br>7 - transitions associated with aging.	1 - Learn about the differentiation/evolutionary processes (bio-psycho-social and spiritual) inherent to aging and their impact on human care needs.<br>2 - understand the need to adapt nursing care to the singularities of the elderly person.<br>3 - learn to care for elderly people by helping them develop their health project and/or respond to and deal with changes resulting from disease processes.<br>4 - acquire relational and technical skills that enable personalized and quality care for elderly people and their caregivers.

		7.1. - evidence-based nursing intervention - transition theory.<br>8 - prevalent disease processes.<br>8.1. - dealing with the changes caused by chronic illness - nurse intervention.<br>8.2 - humanization of institutions.<br>8.3 - monitoring the person at home and supporting caregivers.<br>9 - prevention of complications, accidents, violence and mistreatment.<br>10 - dignification of the end of life and support in mourning.<br>11 - elderly-centered care - integrated and continuous.<br>12 - the nurse and the care team.<br>13 - rights of the elderly and ethical issues linked to aging.	
	Course unit:<br>Elderly care practice I<br>(2nd year/2nd semester – 12 ECTS)	1 - provision of nursing care to the elderly as a human being in transition, interacting with the environment, who seeks well-being and independence in the satisfaction of fundamental needs, both when they feel healthy and when they experience disease processes.<br>2 - planning, execution and evaluation of nursing interventions for the elderly using the nursing process as a scientific work methodology and the taxonomy of the international classification for nursing practice.<br>3 - mobilization of theoretical and theoretical-practical contents capable of supporting the process of caring for the elderly, as a human being in transition interacting with the environment, who seeks well-being and independence in the satisfaction of fundamental needs, both when they feel healthy and when they experience disease processes.	1 - caring for the elderly<br>2 - plan, execute nursing interventions and evaluate outcomes for the elderly using the nursing process as a scientific work methodology and the taxonomy of the international classification for nursing practice and mobilizing theoretical and theoretical-practical contents capable of supporting the care process.
	Course unit:<br>Adult and elderly care practice ii	1 - provision of comprehensive nursing care to adult and elderly medical patients in a hospital	1 - caring for adults and elderly people sick with medical conditions, applying the fundamental

	(2nd year/2nd semester - 18ects)	context, applying the nursing process as a scientific work methodology and integrating the technical, scientific, relational dimension and the values underlying all of the nurse's intervention.<br>2 - provision of comprehensive nursing care to adult and elderly surgical patients in a hospital context, applying the nursing process as a scientific work methodology and integrating the technical, scientific, relational dimension and the values underlying all of the nurse's intervention.	principles of scientific work methodology, in a hospital context and mobilizing theoretical and theoretical-practical content capable of supporting the care process.<br>2 - caring for adults and elderly people suffering from surgical conditions, applying the fundamental principles of scientific work methodology, in a hospital context and mobilizing theoretical and theoretical-practical content capable of supporting the care process.<br>3 - develop technical and relational skills in the practice of nursing care for people suffering from medical and surgical illnesses, in a hospital context.
**Universitat De Les Illes Balears (Spain)**<br><br>**Consulta De Guías Docentes (Uib.E s)**<br><br>**21619. Enfermería En La Persona Mayor (2024-25) - Grado En Enfermería - Oferta - Grado - Aprende - Universitat De Les Illes Balears (Uib.Es)**	Unidade curricular:<br><br>Nursing the elderly<br>(2nd year/2nd semester – 6ECTS)	Thematic unit i: general geronto-geriatric considerations<br>Topic 1. Concepts and generalities related to the aging process.<br>Topic 2. Characteristics and consequences of population aging.<br>Topic 3. Aging and health.<br>Thematic unit ii: social and health care<br>Topic 4. Abuse of older people.<br>Topic 5. Healthcare settings: primary and hospital care.<br>Topic 6. Care areas: socio-health resources.<br>Topic 7. Care for the elderly at home.<br>Topic 8. Promotion of autonomy and care for people in a situation of dependency.<br>Thematic unit iii: comprehensive assessment of the elderly<br>Topic 9. Assessment of the elderly.<br>Thematic unit iv: general care of the daily life of the elderly<br>Topic 10. Nutritional needs. Nutritional assessment.<br>Topic 11. Activity and physical exercise. Gait impairment. Falls.<br>Topic 12. Sexuality in the elderly	Specific:<br><br>• ability to apply the theoretical and methodological principles of nursing acquired in professional practice, considering quality criteria at any stage of the life cycle and considering diversity and multiculturalism.<br><br>• ability to assess, analyse, plan and evaluate health situations susceptible to nursing care in the individual, family and community to develop health education projects regarding disease prevention and promotion. Of the health<br><br>• ability to use and apply scientific, technological and technical knowledge in clinical practice that favours the continuity and complementarity of nursing care.<br><br>Generic<br><br>• analysis and synthesis capacity

		Topic 13. Rest and sleep. Sleep physiology in old age.<br>Topic 14. Pharmacotherapy. Adverse reactions and drug interactions. Adherence to pharmacological treatment. Assistive devices.<br>Topic 15. Immune system and old age. Risk of infection.<br>Topic 16. Urinary and faecal elimination. Functional urinary incontinence. Constipation.<br>Topic 17. Alteration of perception. View. Ear.<br>Topic 18. Pain management in older people.<br>Topic 19. Cognitive problems. Dementia. Alzheimer disease	• ability to identify problems and make decisions based on research results to solve them autonomously.
