# Supplementary material for: Gerontology and Geriatrics in Undergraduate Nursing Education in Portugal and Spain: An Integrative and Comparative Curriculum Review
Source: Healthcare (Basel). 2024 Sep 6;12(17):1786. doi: 10.3390/healthcare12171786 (PMC11395543; doi:10.3390/healthcare12171786)
Supplement: Supplementary file 1 [file healthcare-12-01786-s001.zip › Supplementary_materia_tableS2_extraction tool.pdf]

Supplementary Materials Table S2 – Screening criteria

Author(s) /Year
Local
Title
Study design
Study objective
Results and key findings
