# Supplementary material for: Gerontology and Geriatrics in Undergraduate Nursing Education in Portugal and Spain: An Integrative and Comparative Curriculum Review
Source: Healthcare (Basel). 2024 Sep 6;12(17):1786. doi: 10.3390/healthcare12171786 (PMC11395543; doi:10.3390/healthcare12171786)
Supplement: Supplementary file 1 [file healthcare-12-01786-s001.zip › Supplmentary_material_tableS1_Integrative review search strategy.pdf]

Supplementary Materials Table S1: Integrative review search strategy

Search	Query	Record Retrieved
	**PubMed**	
#1	"nursing"[Subheading] OR "nursing"[MeSH Terms] OR Nursing[Text Word]	55,456 results
#2	"Education, Nursing"[MeSH Terms] OR "Nursing Education" OR ("Curriculum"[MeSH Terms] OR "Educational Programs" AND "Nursing" [MeSH Terms])	5,204 results
#3	"geriatric nursing"[MeSH Terms] OR Geriatric Nursing[Text Word] OR "Gerontologic Nursing" OR "Geriatric Care"	1,212 results
#4	"Gerontology education" OR "Education in aging" OR "Gerontology studies" OR "geriatric studies"	221 results
#5	**#1 + #2 + #3 +#4**	**54 results**
	**CINAHL (via EBSCOhost)**	
#1	"nursing"	205,269
#2	"Nursing education" OR "Nurse training" OR "Nurse education programs" OR "Nursing curriculum" OR Nursing syllabus" OR Nursing programmes"	9,305
#3	"Geriatric nursing" OR "Gerontologic nursing" OR "Geriatric care"	3,418
#4	"Gerontology education" OR "Education in aging" OR "Gerontology studies" OR "geriatric studies"	38
#5	**#1 + #2 + #3 + #4**	**25**
	**Medline (via EBSCOhost)**	
#1	"nursing"	124,099
#2	"Nursing education" OR "Nurse training" OR "Nurse education programs" OR "Nursing curriculum" OR Nursing syllabus" OR Nursing programmes"	5,302
#3	"Geriatric nursing" OR "Gerontologic nursing" OR "Geriatric care"	1,730
#4	"Gerontology education" OR "Education in aging" OR "Gerontology studies"	145
#5	**#1 + #2 + #3 + #4**	**4**
	**Nursing Allied (via EBSCOhost)**	
#1	"nursing"	112,303
#2	"Nursing education" OR "Nurse training" OR "Nurse education programs" OR "Nursing curriculum" OR Nursing syllabus" OR Nursing programmes"	5,853
#3	"Geriatric nursing" OR "Gerontologic nursing" OR "Geriatric care"	638

**#4**	'Gerontology education" OR "Education in aging" OR 'Gerontology studies"	38
**#5**	**#1 + #2 + #3 + #4**	**3**
**Filters Applied**	**Language: English, Portuguese, Spanish. Integral text.**	
