# Supplementary figures and images for: Gerontology and Geriatrics in Undergraduate Nursing Education in Portugal and Spain: An Integrative and Comparative Curriculum Review
Source: Healthcare (Basel). 2024 Sep 6;12(17):1786. doi: 10.3390/healthcare12171786 (PMC11395543; doi:10.3390/healthcare12171786)

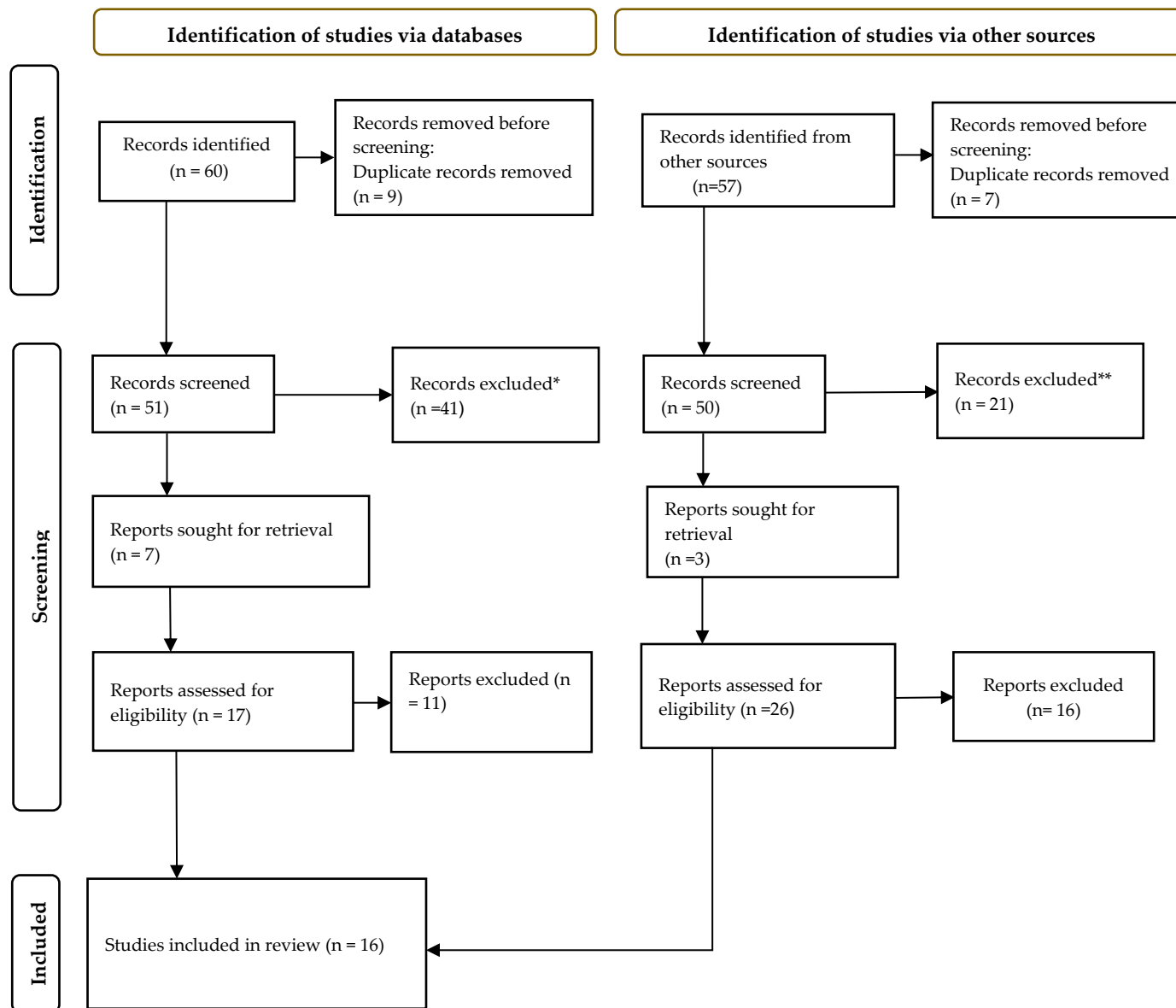

Supplement: Supplementary file 1 [file healthcare-12-01786-s001.zip › Supplementary_material_, Figure S1 PRISMA Integrative review results.pdf]
